# Assessing Risk and Preventing 30-Day Readmissions in Decompensated Heart Failure: Opportunity to Intervene?

**DOI:** 10.1007/s11897-015-0266-4

**Published:** 2015-08-20

**Authors:** Richard Dunbar-Yaffe, Audra Stitt, Joseph J. Lee, Shanas Mohamed, Douglas S. Lee

**Affiliations:** Institute for Clinical Evaluative Sciences, University of Toronto, 2075 Bayview Avenue, Room G-106, Toronto, ON M4N 3M5 Canada; Peter Munk Cardiac Centre, University Health Network, Toronto, Ontario Canada; Peter Munk Cardiac Centre and Joint Department of Medical Imaging, University Health Network, Toronto, Ontario Canada; Institute for Health Policy, Management, and Evaluation, University of Toronto, Toronto, Ontario Canada

**Keywords:** Heart failure, Readmission, Hospital, Prevention, Risk prediction, Outcomes, Health services delivery, Health policy

## Abstract

Heart failure (HF) patients are at high risk of hospital readmission, which contributes to substantial health care costs. There is great interest in strategies to reduce rehospitalization for HF. However, many readmissions occur within 30 days of initial hospital discharge, presenting a challenge for interventions to be instituted in a short time frame. Potential strategies to reduce readmissions for HF can be classified into three different forms. First, patients who are at high risk of readmission can be identified even before their initial index hospital discharge. Second, ambulatory remote monitoring strategies may be instituted to identify early warning signs before acute decompensation of HF occurs. Finally, strategies may be employed in the emergency department to identify low-risk patients who may not need hospital readmission. If symptoms improve with initial therapy, low-risk patients could be referred to specialized, rapid outpatient follow-up care where investigations and therapy can occur in an outpatient setting.

## Introduction

Heart failure is a major health problem in the developed world representing a substantial portion of emergency department (ED) presentations and admissions, and it is a leading reason for hospitalizations globally [1]. Clinical deteriorations leading to ED visits and hospital admissions contribute substantially to the more than $108 billion in health care expenditures for heart failure worldwide [2].

Patients who survive their index admission are also at significant risk for readmission. With 30-day readmission estimates of nearly 25 % [3] and 6-month readmission rates as high as 46 % [4], heart failure is the leading reason for hospitalizations among the elderly. There has been great interest in the concept of preventing readmissions among patients with heart failure (HF), which has been accelerated further because hospital-level performance measures on 30-day readmissions have been linked to financial penalties in the USA [5].

There are three major types of interventions and strategies that may be employed to lead to a reduction in HF readmissions, and different settings in which they may be deployed. First, the earliest time point when a strategy for reducing readmissions can be initiated is prior to discharge home from hospital. Second, once patients are in the community, they can be monitored closely such that potential decompensations might be identified earlier allowing the opportunity to intervene and provide timely care. Third, the final opportunity to intervene to reduce readmissions is in the ED itself where there may be opportunities to divert patients away from inpatient-based care.

In this review, we will discuss how interventions at each of these phases could potentially be utilized to reduce 30-day readmissions among patients with heart failure. Recent attempts to curb high readmission rates through efforts such as disease management programs, education initiatives, and biomarker-guided therapy will also be discussed.

## Identifying Patients at Risk for Readmission

The first opportunity to prevent readmission occurs before the patient is discharged from hospital, by the identification of patients who are at highest risk of readmission. The prediction of readmission risk begins before the initial discharge because readmissions can occur even within the first day of discharge. Dharmarajan et al*.* reported that over 60 % of all 30-day HF readmissions occurred within 15 days of initial hospital discharge [6]. Therefore, substantial effort has been devoted to predict which patients are at risk for readmission, because identifying those at highest risk of readmission would allow the maximum allocation of resources to be directed toward keeping them well at home and out of hospital.

While there have been many published studies aiming to predict the risk of 30-day readmissions among patients with HF, there are also substantial variations in the characteristics that have been associated with a heightened readmission risk. A recent systematic review by Ross and colleagues thoroughly examined literature sources until 2007, examining 112 studies of various designs and statistical methodologies that examined patient-level characteristics associated with readmission [7]. While age and sex were the most commonly included variables in readmission models (>70 % of studies examined), there was no consistent association among the diverse array of covariates. Comorbidity measures frequently included conditions such as diabetes mellitus and hypertension but were also not consistently associated with readmission. The biomarker B-type natriuretic peptide, the product of a neurohormonal cascade related to ventricular stretch, was one of the laboratory tests whose elevation was consistently associated with readmission (17 of 24 studies containing the variable reported a significant association). Elevated troponin levels were also consistently associated with increased readmission risk.

In order to build upon the results of Ross et al., we conducted an updated literature search reexamining the potential predictors associated with readmission risk. RCT cohorts, prospective cohorts, and retrospective analyses of either hospital-level or administrative data (i.e., Medicare) were used as evidence sources. We examined a number of outcomes including all-cause and HF-related readmissions, and composite outcomes with death.

### Demographics

Among the demographic features, age has been inconsistently associated with HF readmission [8–11], while there was no significant association between sex and readmission risk in any of the data sources examined [8, 10, 12–18]. The patient’s race was associated with readmission risk, with non-Caucasian patients being most frequently readmitted [10, 13, 15, 19]. While comparison of patients based on their insurance status does introduce some degree of confounding, it is notable that those with Medicare/Medicaid coverage exhibited higher risk of readmission [11–13, 18, 20]. Patients of lower socioeconomic status were often found to be at higher risk of readmission [13, 14, 20, 21].

### Comorbidities

While comorbidities, such as hypertension, renal failure, chronic obstructive pulmonary disease, peripheral vascular disease, electrolyte abnormalities, cerebrovascular disease, cancer, and diabetes mellitus, have been commonly studied, they are inconsistently associated with readmission when examined independently [8, 9, 12, 15, 17, 22]. However, comorbidities clearly are linked with readmission risk because composite indices such as the Charlson score were associated with increased HF readmission risk [8, 9]. Furthermore, while psychiatric conditions are increasingly prevalent, comorbidities such as depression have not been found to be consistently associated with readmission [9, 12–14, 18, 23–30].

Shorter length of stay during the index hospitalization and more prior hospitalizations were associated with increased risk of readmission [8, 10, 11, 15]. The former association suggests that HF patients with shorter length of stay may be at greater risk of readmission because they have been inadequately decongested. The latter finding suggests that hospitalization itself may predispose patients to a state of frailty, which begets further hospitalization.

### Novel Factors

Biomarkers have been found to be associated with higher readmission risk, including B-type natriuretic peptide [31–37] and elevated troponin levels [35, 37–40]. Cystatin C, a relatively new biomarker associated with renal function, is also significantly associated with readmission risk [35, 41–43].

## Modeling Readmission Risk

Predictive models using administrative or clinical data sources have great potential utility. Ross and colleagues identified five studies [44–48] whose primary purpose was generation of a predictive model [7]. The majority of studies utilized retrospective data from either administrative databases or randomized controlled trial cohorts. There was substantial variability in the number of patients involved (ranging from 257 to 42,731), follow-up duration (60 days to 1 year), and variation in outcomes from hospital readmission, composite of readmission or death, or heart-failure-specific readmission. The c-statistics provided in three of the five studies was modest, ranging from 0.60 and 0.69.

Our review identified an additional five studies modeling heart failure readmission risk. Keenan and colleagues [49] developed an administrative claims model approved by the National Quality Forum to estimate hospital-specific readmission rates for Medicare patients hospitalized with heart failure. Both heart failure hospitalization and candidate model variables were based on the presence of ICD-9 diagnostic codes within the 12 months prior to index hospitalization. Of 189 candidate variables assessed for inclusion, 37 (2 demographic, 9 cardiovascular, 26 comorbidity) were included in the final model, which was developed using multiple logistic regression analysis. With 567,447 heart failure hospitalizations examined, the c-statistic of their proposed model was 0.61.

Amarasingham and colleagues [13] developed a novel model using routinely available admission variables that are present in electronic medical records within the first 24 h of hospital presentation. This retrospectively examined cohort of 1372 patients at a single center, Parkland Memorial Hospital in Dallas, Texas, was identified using a principal admission diagnosis code of heart failure. The crude 30-day readmission rate was 24.7 %, and the model c-statistic was 0.72. Two important features distinguish this model from others. First, their model depends on easily attainable administrative data available generally at the time of admission. Second, the group identified important markers of social behavior that could indicate a fragile social situation prone to readmission risk. These markers included the number of address changes in the past year, number of ED visits in the past year, and a history of confirmed cocaine use.

To supplement the claims-based model, Hammill et al*.* sought to incorporate clinical variables with the aim of improving discriminatory ability [50]. They obtained clinical data of patients aged ≥65 years from the Get With The Guidelines–Heart Failure (GWTG-HF) registry linked to Medicare claims databases. Excluding stays ≤1 day and elective hospitalizations, the observed 30-day readmission rate was 21.9 % and the mortality rate was 10.5 %. While the “claims-clinical” model included additional variables, such as left ventricular ejection fraction, heart rate, hemoglobin, creatinine, serum sodium, systolic blood pressure, and weight, the c-statistic for predicting readmissions was not significantly improved compared to the claims-only model.

The length of stay, acuity, Charlson comorbidity, and emergency utilization (LACE) score was developed to assess readmission risk for acute coronary syndromes and cancer diagnoses with a highly discriminative c-statistic of 0.77 [51]. Au et al. sought to validate the model in patients with heart failure using administrative databases in Alberta, Canada [8]. They evaluated the model in nearly 60,000 discharged patients and compared it with several other models including the model proposed by Keenan et al. The primary outcome of death and readmission at 30 days was determined to be 18.7 % in this cohort, with 5.1 % mortality and 15.9 % all-cause readmission rate. The c-statistics of the models and variants ranged from 0.55 to 0.61.

A summary demonstrating the wide variability of covariates in different clinical models for readmission risk assessment is shown in Table 1.

**Table 1:** Clinical prediction model covariates for HF readmission

	Felker [45]	Krumholz [46]	Amarasingham [13]	Van Walraven [51]
Demographic characteristics				
Age	x		x	
Sex			x	
Marital status			x	
Low SES			x	
# Home address changes			x	
Medicare			x	
Cardiovascular status				
NYHA class	x			
Prior HF		x		x
Prior MI				x
Health services use history				
Prior admission/ED visit		x	x	x
Prior missed clinic visit			x	
Pharmacy use			x	
Acute presentation features				
Daytime ED presentation			x	
Acute admission				x
Length of stay				x
Vital signs				
Systolic BP	x		x	
Diastolic BP			x	
Heart rate			x	
Temperature			x	
Laboratory tests				
Serum sodium	x		x	
Blood urea nitrogen	x		x	
Creatinine		x	x	
WBC count			x	
Albumin			x	
CK			x	
Troponin			x	
INR			x	
Bilirubin			x	
Arterial pH			x	
Arterial pCO_2_			x	
Comorbid conditions				
Diabetes		x		x
COPD			x	x
Cancer			x	x
Peripheral vascular disease				x
Cerebrovascular disease				x
Liver disease				x
Connective tissue disease				x
HIV infection				x
Mental health				
Depression/anxiety			x	
Altered mental status			x	
Cocaine abuse			x	
Dementia				x

*SES* socioeconomic status; *NYHA* New York Heart Association; *HF* heart failure; *MI* myocardial infarction; *ED* emergency department; *BP* blood pressure; *WBC* white blood count; *CK* creatine kinase; *COPD* chronic obstructive pulmonary disease

## Ambulatory Strategies

Several ambulatory strategies exist with the goal of keeping patients out of hospital, thereby preventing readmissions. One such strategy is a disease management program. Originally described by Rich et al. [52], a wealth of randomized trials provide support for this strategy, leading to a reduction in readmissions longitudinally over time. A Cochrane review by Takeda and colleagues identified 25 randomized controlled trials with nearly 6000 total participants classified into case management interventions, clinical interventions, and multidisciplinary interventions [53]. The authors found that case management interventions significantly reduced heart-failure-related readmissions at both 6 (odds ratio 0.64) and 12 months (odds ratio 0.47), but there was less significant impact on all-cause readmissions at 12-month (odds ratio 0.75) follow-up [53]. Interestingly, in a propensity-matched analysis, Wijeysundera et al*.* found that care provided in multidisciplinary heart failure clinics was associated with reduced mortality but higher readmission rates [54], suggesting that the two outcomes need to be considered separately when designing a strategy to reduce readmissions. While effective longitudinally, the multidisciplinary heart failure clinic strategy has not specifically been applied to the transitional care setting to reduce 30-day readmissions.

Patients with heart failure may also be readmitted to hospital with decompensations that may have premonitory warning signs. Earlier detection of physiological abnormalities can be achieved using technologies such as remote telemonitoring devices. Telemonitoring interventions can allow measurement of vital signs, weight, and symptoms “at a distance,” and more advanced devices offer oximetry, GPS, and accelerometry data which can be transferred to a central server for monitoring. Prior studies have shown that higher heart rates in the peri-discharge transitional period have significant prognostic implications for HF patients with an increased risk of HF and cardiovascular hospitalizations with heart rates >90 beats/min [55]. In addition, higher and lower blood pressure peri-discharge has been demonstrated to be associated with increased mortality risk [56].

While technologies are available for remote monitoring in the transitional care period, its role in the pathway of HF patients after hospital discharge is unclear. This is, in part, due to the variable results in the published literature, which demonstrates heterogeneity. Programs vary in the intensity of the intervention, how telemonitored data are treated, and the degree of patient involvement in obtaining measurements at home. While an in-depth evaluation of telemonitoring interventions is outside of the scope of this review, some patterns are evident.

First, remote monitoring of symptoms or physiological measurements that were less intensive and had little clinical involvement by nurse and/or physician was generally not effective. The intensity of the intervention in these negative studies were demonstrated by non-daily ascertainment of physiologic measurements or symptoms [57, 58], lack of nurse involvement in the intervention [59], lack of nurse review on weekends [60, 61], or absence of highly engaged HF specialist physician response team [62–64], compared to studies with positive results favoring telemonitoring [65–69]. One of the trials that did not demonstrate a significant benefit was a large trial of telemonitoring, which enrolled 1653 patients [61]. In this study, daily phone calls were made to an automated voice system, and data were reviewed on weekdays by site coordinators. There was no significant difference between telemonitoring and usual care groups in the composite of death and readmission at 180 days. Although it is the largest trial to date, many cite low adherence (14 % of patients never activated their telemonitoring and only 55 % of patients were adherent throughout the trial period) as an additional caveat in interpretation.

In contrast, several studies illustrate highly successful examples in the spectrum of telemonitoring interventions. Giordano and colleagues [70] offered a unique intervention in which constant transmission of vital data was supplied through a mobile phone to a monitoring station for 460 patients in Italy. Nurses received the data and interacted closely with treating physicians such that only the physician could recommend an emergency room visit. A reduction in 1-year readmission was demonstrated (relative risk 0.56; 95 % confidence interval 0.39, 0.84) with a subsequent mortality benefit and reduction in mean cost of readmission [70].

In Spain, Atienza et al. conducted a randomized trial of telemonitoring in 338 patients, where the intervention began prior to hospital discharge [71]. In the pre-discharge phase, a cardiac nurse emphasized disease knowledge and self-care to hospitalized patients. In addition to daily remote vital signs transmission, patients were seen by their primary care provider within 2 weeks and received regular follow-up in the heart function clinic. Their intervention was highly successful, demonstrating a significant reduction in events per observation year and readmissions per patient year. Furthermore, a nearly twofold reduction in hospital days and a significant mortality reduction were shown. The importance of specialist involvement was demonstrated in a telemonitoring study conducted in the USA by Laramee et al.[58] While the primary intervention (in-hospital discharge planning, diet and fluid recommendations, and self-monitoring followed by a telephone intervention in which participants were called every 1 to 3 days with respect to their symptoms) did not significantly reduce readmission at 90 days, there was a significant impact in the subset of patients whose cardiologists were more reachable by the surveying nurse (HF readmission rate 2 vs. 14 %), presumably due to improved physician response to symptomatic changes [58].

## Emergency-Department-Based Strategies to Reduce Readmissions

The final point where intervention is possible to reduce readmissions is in the emergency department. We have previously shown that some patients who present to the ED have a low risk of mortality [72]. While some of these patients may have symptoms or other considerations that might require hospital admission, some low-risk patients can be discharged home to receive outpatient care. If these low-risk patients improve symptomatically after diuresis while being observed in the ED, they can be discharged home and a potential readmission may be averted. To implement this strategy, several components of care must be available.

**Fig. 1:**
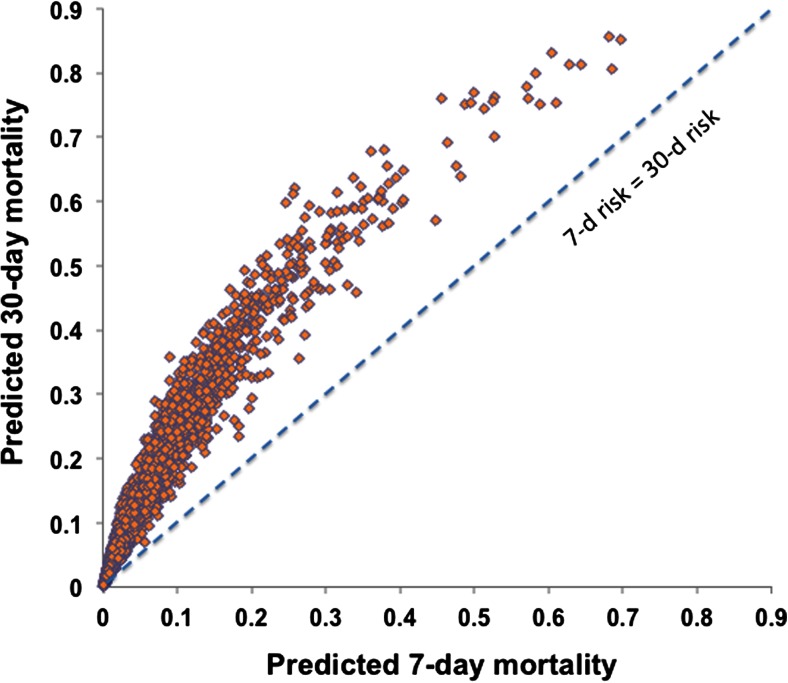
Simultaneous estimation of 7-day and 30-day mortality risks using the EHMRG algorithms

First, a validated risk stratification algorithm must be available. We have developed the Emergency Heart Failure Mortality Risk Grade (EHMRG), a clinical risk algorithm that stratifies risk of 7-day mortality which was derived and validated in >12,000 patients presenting to the ED (https://ehmrg.ices.on.ca) [73]. The predictive ability of the EHMRG has been extended to predict 30-day mortality by the addition of one more variable, the presence or absence of ST segment depression on the 12-lead electrocardiogram [74]. The addition of ST segment depression resulted in a net reclassification improvement of 17 % compared to the EHMRG 7-day model alone, for prediction of 30-day mortality risk [74]. The composite of these two risk scores provides a simultaneous estimate of risk at two different points in time (Fig. 1), which we have termed the EHMRG30-ST model. The ability to determine that patients are not high risk of mortality at an early time and at 30 days is important if outpatient ambulatory care will be provided during the transition from hospital to home, which extends up to 30 days post-ED discharge.

Second, the ability to observe the patient before making the final decision to admit or discharge the patient is beneficial. This may occur in an observation unit setting, and it is important because it allows physicians to determine if there has been a response to diuretics. Patients who are low risk and have responded to diuretics are good candidates for discharge home. In contrast, patients who are low risk but remain congested after initial diuretic administration may still require an admission for further diuresis. These patients could potentially be admitted for a short-stay admission and be rapidly discharged with outpatient follow-up. During this period of observation, troponin measurement could be repeated and the patient could be further monitored. Given the importance of troponin in prognosis [38], if there is an increase in troponin into an abnormal range, this might lead the medical team to admit the patient for investigation if the ischemic disease status of the patient is unknown. Other considerations that may become apparent during the period of observation, including significant deviations of vital signs, may also lead to the decision to admit rather than discharge home [75].

**Fig. 2:**
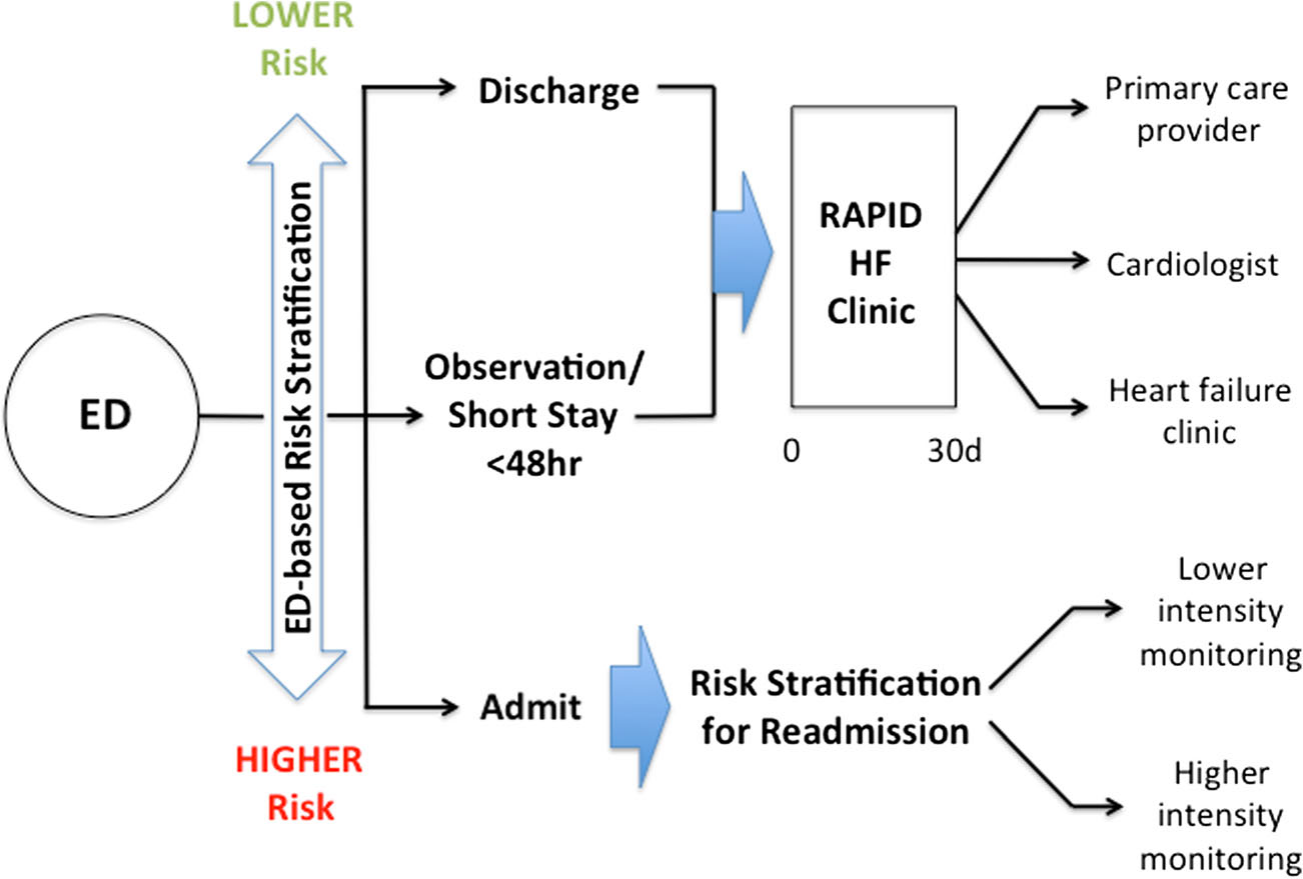
HF risk stratification and decision-making after emergency department presentation, admission, and hospital discharge

Third, the ability to follow patients rapidly after discharge is critically important to this strategy. We have shown that cardiac specialist care leads to substantial improvement in survival and readmission-free survival in patients who are discharged from the ED [76]. The Rapid Ambulatory Program for Investigation and Diagnosis of HF (RAPID-HF) provides specialized follow-up care to patients who are discharged from the ED or observation unit, or after a short-stay hospital admission (<48 h) in a transitional care clinic (Fig. 2). Ideally, patients should be followed in the RAPID-HF clinic within 48 h from index discharge to ensure that they have responded well to diuretic therapy and to determine if any adjustment is required. In addition, echocardiography to determine the underlying left ventricular systolic function, and laboratory testing to reevaluate electrolytes and renal function should also be performed. If the presence of ischemic heart disease is unknown, testing may be performed using myocardial perfusion imaging or coronary angiography, for etiologic evaluation. The RAPID-HF clinic is a transitional care clinic that provides care for a maximum of 30 days, after which time, longitudinal care is transferred to a cardiac specialist or a multidisciplinary HF clinic. Therefore, the care provided in the RAPID-HF clinic moves the diagnostic and therapeutic interventions, which in the past might have been performed in hospital, into the ambulatory care setting.

## Conclusion

Readmissions among heart failure patients after a recent hospital discharge are influenced by multiple potential factors. While readmissions can occur at any time, there is particular interest in events occurring within 30 days—a short time period in which to implement interventions to reduce readmissions. Interventions that have been shown to have greater impacts on reducing readmissions tend to be more intensive in terms of the frequency and type of monitoring, the engagement of nurses, and involvement of more specialized cardiac care in treatment decisions. There does not seem to be a single component that drives benefit, because as the degree of the intervention becomes less intense, the benefits are attenuated. It is for this reason that we need better ways to identify those heart failure patients who are at the highest risk of readmission. Specifically, the resources necessary to reduce readmissions are likely too costly to apply to all patients but are likely to be most efficiently applied if patients who are at highest risk and most likely to benefit can receive the highest-intensity interventions.

Identifying those at high risk of hospital readmission, however, has been a challenge because of the very modest predictive capability of most algorithms to identify high-risk subsets. In addition, the competing risk of readmissions with death poses another major challenge to risk stratification modeling. Specifically, characteristics that lead to high rates of death may be associated with lower readmission risk if competing risks are not accounted for in the analysis [3]. Indeed, reducing readmissions as a consequence of higher mortality is clearly an undesired result. Consequently, there are analytical challenges because readmission and death might not be readily combined, as these two types of events are not equal in severity to justify combining them into a single composite endpoint. While challenging, it remains important to continue our efforts to develop risk stratification models for readmission in order to target interventions and quality improvement strategies in the most efficient way.

Finally, HF patients who seek care in the emergency department have varying disease severity, and not all patients who present within 30 days after initial hospital discharge need to be readmitted. The ability to identify those who are low risk of mortality is important so that these patients can be managed without hospitalization in an ambulatory care setting that can ensure rapid and specialized follow-up care. Patients need to be at low risk of death to ensure that adverse events will not occur while the transitional care clinic provides the necessary diagnostic tests and therapies that can improve symptoms and prognosis and ultimately reduce the downstream risk of hospital readmission.
